# Pseudomonas serbiensis sp. nov. isolated from watermelon and muskmelon in Serbia

**DOI:** 10.1099/ijsem.0.006613

**Published:** 2024-12-18

**Authors:** Kiersten R. Fullem, Aleksa Obradović, Michelle P. MacLellan, Mousami Poudel, Gerald V. Minsavage, Erica M. Goss, Neha Potnis, Jeffrey B. Jones, Mathews L. Paret

**Affiliations:** 1 Department of Plant Pathology, University of Florida, Gainesville, FL, USA; 2 Plant Pathology Department, Faculty of Agriculture, University of Belgrade, Belgrade, Serbia; 3 Department of Plant Pathology, University of Georgia, Tifton, GA, USA; 4 Department of Entomology and Plant Pathology, Auburn University, Auburn, AL, USA

**Keywords:** bacteria, cucurbits, *Pseudomonas*, watermelon

## Abstract

Six *Pseudomonas* strains isolated from muskmelon and watermelon seedlings affected by stem rot and wilting in Serbia were reported as *P. cichorii* based on pathogenicity, LOPAT and cell wall fatty acid analyses. Recent bacterial isolates from cucurbit crops displaying *P. cichorii*-like symptoms in Alabama, USA, were identified as *P. capsici*, prompting polyphasic re-evaluation of the Serbian strains. All six strains were found to cause severe disease in watermelon and squash seedlings under greenhouse conditions. Strains KFB 138 and KFB 140 underwent whole-genome sequencing and were found to have the highest level of 16S rRNA similarity to *P. lijiangensis* LJ2^T^ (both 99.87%). Phylogenies based on housekeeping genes and core-genome analysis placed both strains into phylogroup 11 of the *Pseudomonas syringae* species complex, with KFB 138 forming a lineage basal to all other phylogroup 11 members. In core-genome phylogeny, KFB 140 was placed into a clade alongside *P. lijiangensis* LJ2^T^. Average nucleotide identity based on blast (ANIb) identified KFB 140 as a member of *P. lijiangensis* (95.85%), though KFB 138 did not produce an ANIb value over 95% to any *Pseudomonas* type strain to which it was compared. Values for *in silico* DNA–DNA hybridization for both strains were below 70% to all reference strains tested, though KFB 140 was found to be most similar to * P. lijiangensis* (68.2%). KFB 138 and KFB 140 were further characterized using the online Type Genome Server, biochemical profiling with the Biolog Gen III MicroPlate system, matrix-assisted laser desorption/ionization time of flight mass spectrometry and imaged with transmission electron microscopy. From the results of the above analyses, we conclude that KFB 140 is a member of the species *P. lijiangensis* and that KFB 138 represents a novel *Pseudomonas* species, for which we propose the name *Pseudomonas serbiensis* (KFB 138^T^, NCPPB 4762=LMG 33366), named for its location of isolation.

## Introduction

*Pseudomonas* is the largest of all Gram-negative bacterial genera, currently containing over 330 validly named species and rapidly growing, with 20 new species added in 2023 alone [1,2]. The genetic and physiological diversity of pseudomonads allows them to occupy a vast array of niches, including plants, animals, fungi, fresh and salt water, and soils [3,5]. They are capable of living freely in the environment, as well as forming close beneficial, commensal, and parasitic relationships with other organisms, and may switch between lifestyles [4,6,8]. The genus contains many species that are responsible for significant diseases of plants, fungi and animals, including humans [3,6, 9]. Unsurprisingly, given the ubiquity and adaptability of the genus, nearly all vascular plants are affected by pathogenic *Pseudomonas* species, which cause a wide variety of disease symptoms [3,7, 9].

*P. cichorii*, first isolated from chicory (*Cichorium intybus* and *C. endivia*) in 1925, possesses an extensive host range, including numerous economically significant agricultural and ornamental crops, such as lettuce (*Lactua sativa*), tomato (*Solanum lycopersicum*), basil (*Ocimum basilicum*), chrysanthemum (*Chrysanthemum* spp.) and geranium (*Pelargonium hortorum*) [10,15]. Symptoms of *P. cichorii* infection vary by host, but typically include necrotic and/or water-soaked lesions, which are commonly seen on foliage and may also be present on petioles, stems and flower buds [11,12, 14,16]. These lesions are often reported to contain concentric rings [17,21], and, under humid conditions, they may grow and coalesce, creating large areas of blighted or rotting tissue [11,21, 22]. *P. cichorii* is also associated with stem infections, with symptoms including stem lesions, rots, discolouration of stem and vascular tissues and, in the case of severe infections, blighting and wilting [11,12, 21, 23, 24].

While disease outbreaks associated with *P. cichorii* are commonly reported, identification of isolates is often based on phenotypic assays, such as fluorescence and LOPAT testing [levan production, oxidase activity, pectolytic activity, arginine dihydrolase production and hypersensitive response (HR) on tobacco], and minimal sequence data, typically only including analysis of the 16S rRNA gene sequence and sometimes pathogenicity gene cluster *hrcRST* [17,24,28]. Though 16S rRNA sequencing is widely used for prokaryotic species identification, it lacks the discriminatory power to distinguish between closely related species [1,29,31]. Whole-genome sequence comparisons, including average nucleotide identity (ANI) and DNA–DNA hybridization (DDH), offer a higher level of precision for bacterial identification and can differentiate species indistinguishable by 16S rRNA analysis [1,29, 32].

Recent studies utilizing whole-genome genetic analyses have identified novel plant-pathogenic *Pseudomonas* species, including *P. capsici* and *P. lijiangensis*, which are phenotypically and genetically similar to *P. cichorii*, and have also revealed that many bacterial isolates, previously reported as *P. cichorii*, in actuality belong to these distinct species [33,35]. *P. capsici* was first described from pepper seedlings (*Capsicum anuum*) displaying dark, water-soaked foliar lesions in 2019 in Georgia, USA [33], while *P. lijiangensis* was initially described in 2019 in China from dark foliar lesions on tobacco (*Nicotiana tabacum*) [34]. In these descriptions, strains of *P. capsici* and *P. lijiangensis* were characterized using phenotypic assays, 16S rRNA gene sequence analysis, average nucleotide identity based on blast (ANIb) and *in silico* DNA–DNA hybridization (isDDH). In both studies, whole-genome comparisons effectively distinguished *P. capsici* and *P. lijiangensis* strains from *P. cichorii*, while phenotypic assays and 16S rRNA results were insufficient [33,34].

A 2023 study characterizing *P. capsici* isolates from pepper and tomato plants using whole-genome analyses revealed that many bacterial strains isolated from diverse locations, including the United States, Barbados and Brazil, which had previously been reported as *P. cichorii*, in actuality belonged to *P. capsici* [35]. A 2020–2022 survey of pseudomonads associated with bacterial leaf spot of cucurbits in the southeastern United States resulted in the isolation of nine bacterial strains from diseased cucumber (*Cucumis sativus*), squash (*Cucurbita pepo*) and watermelon (*Citrullus lanatus*) plants in Alabama [32]. These strains caused severe disease upon re-inoculation into watermelon and squash seedlings with symptoms reminiscent of *P. cichorii*, including large, dark, water-soaked foliar lesions containing concentric rings and vascular discolouration on cotyledons. Fluorescence and LOPAT results for these strains were also consistent with those of *P. cichorii* [32,36]. Previous reports of *P. cichorii* infecting cucurbits were rare [16] and all Alabama isolates were eventually identified as members of *P. capsici* [32]. These findings prompted the re-examination of six *Pseudomonas* strains isolated from outbreaks of wilt and stem rots in muskmelon (*Cucumis melo*) and watermelon in Serbia that were previously identified as *P. cichorii* using results from fluorescence and LOPAT assays, as well as cell wall fatty acid profiling [37].

## Methods and results

In this study, we describe a polyphasic approach for the identification and characterization of the six *Pseudomonas* strains from Serbia, including phenotypic characterization with fluorescence, LOPAT and pathogenicity assays; biochemical profiling with the Biolog Gen III MicroPlate system; matrix-assisted laser desorption/ionization time of flight mass spectrometry (MALDI-TOF MS); and imaging of cells with transmission electron microscopy (TEM). Furthermore, the strains were subjected to genetic characterization based on 16S rRNA sequence analysis, phylogenies based on multi-locus sequence analysis (MLSA) and core-genome analysis, ANIb and isDDH, including comparison to the Type Genome Server (TYGS).

### Phenotypic assays

Cultures of the six Serbian strains were kindly provided by Dr Aleksa Obradović of the University of Belgrade. The three strains had been isolated in May of 1989 from diseased muskmelon seedlings in Srem, Serbia (KFB 136, KFB 137, KFB 138), and three in June of 1997 from diseased watermelon seedlings in Smederevska Palanka, Serbia (KFB 139, KFB 140, KFB 141). Similar symptoms had been observed in both disease outbreaks, including water-soaked lesions on stems, undersides of cotyledons and true leaves; widespread necrosis; wilting; and softening and collapsing of stems [37]. All six strains were fluorescent under UV light when grown on King’s Medium B (KMB) and Gram-negative according to a standard potassium hydroxide (KOH) assay [38]. LOPAT testing was conducted, including assays for levan production, oxidase activity, pectolytic activity on potato, arginine dihydrolase production and HR, performed on both tobacco and tomato (Table 1). Identical results were observed for all strains tested, with negative results for levan production, pectolytic activity, arginine dihydrolase production and HR on tobacco and positive results for oxidase activity and HR in tomato.

**Table 1.** Tests for levan production, oxidase activity, pectolytic activity, arginine dihydrolase production and HR in both tobacco and tomato (LOPAT) for strains described in this study and representative strains of closely related *Pseudomonas* species

	*P. serbiensis*	*P. lijiangensis*		*P. cichorii*	*P. capsici*	
Assay	KFB 138^T^	KFB 140	LJ2^T^	n/a	19-1^T^	AS1
Levan production	−	−	nr	−	−	−
Oxidase activity	+	+	+	+	+	+
Pectolytic activity	−	−	nr	−	+	−
Arginine dihydrolase activity	−	−	−	−	−	−
Tobacco HR	−	−	nr	+	+	nr
Tomato HR	+	+	nr	nr	nr	+
**Source**	This article	This article	[34]	[38]	[33]	[32]

nr designates a result which was not reported. Strain AS1 is included as a representative of the nine *P. capsici* strains obtained from cucurbits in Alabama, which prompted the research described in this article [33].

## Pathogenicity assays

Pathogenicity of the strains was assessed on watermelon (var. Prisca) and summer squash (*Cucurbita pepo* subsp. *pepo*, var. Conqueror III) seedlings. Seedlings were grown from seed under greenhouse conditions (day temperature = 28 °C, night temperature = 24 °C) with daily watering until 4 weeks of age. For each crop, treatments contained three replicates, each consisting of a single pot containing three seedlings. Plants used for experimental treatments were spray-inoculated with bacterial suspensions adjusted to 10^8^ CFU ml^−1^ until runoff and placed into moistened polyethylene bags, where they remained for 72 h under greenhouse conditions. *P. capsici* strain AS1, known to cause severe *P*. cichorii-like lesions on cucurbit plants, was used as a positive control. Negative controls consisted of completely untreated and unbagged plants, plants that were mock-inoculated with sterile tap water and left unbagged, and mock-inoculated plants that were placed into moistened polyethylene bags. After 72 h, bags were removed, and similar severe disease symptoms were recorded for all experimental treatments. Positive control strain AS1 also produced severe disease on both hosts, and no symptoms were observed in any of the negative control treatments. On watermelon, symptoms produced by Serbian strains included large, tan or brown, water-soaked lesions on cotyledons and true leaves, with some lesions containing concentric rings. On squash, widespread chlorosis was observed on cotyledons, accompanied by large, brown, water-soaked lesions. True leaves contained smaller tan or brown lesions, with or without concentric rings and surrounded by chlorotic halos.

## Whole-genome sequencing and 16S rRNA sequence analysis

As strains were identical in their fluorescence, LOPAT and pathogenicity results, one representative strain from each disease outbreak was selected for further characterization (KFB 138 and KFB 140). DNA extraction was performed using the Wizard Genomic DNA Purification kit (Promega) and resulting DNA was sent to the Microbial Genome Sequencing Center (Pittsburgh, PA) for whole-genome sequencing using the Illumina NextSeq 2000 platform. Genetic reads were assembled using a pipeline containing genome assembly tool SPAdes (v. 3.10.1) [39]; genome statistics for resultant assemblies can be found in Table 2. The 16S rRNA gene sequences for both strains were compared using BLASTn against the National Center for Biotechnology Information’s (NCBI) nucleotide database as well as a database containing 16S rRNA sequences from type strains of all *Pseudomonas* species that had been validly published at the time of analysis and for which genetic sequence data were available (LPSN, *n* = 329) [2]. Both KFB 138 and KFB 140 were found to have the highest percent identity of the 16S rRNA gene sequence to *P. lijiangensis* (strain JL2^T^, accession number GCF_018968705.1) with identical scores of 99.87%.

**Table 2.** Genome statistics, including genome coverage, genome length, N50 value and G+C content for *P. serbiensis* and *P. lijiangensis* strains

	*P. serbiensis*	*P. lijiangensis*
	KFB 138^T^	KFB 140
Genome coverage	80	74
Genome length (Mb)	6.22	6.12
N50 value	232520	248778
G+C content (mol%)	57.50	58.51
Number of Contigs	48	59
Completeness (%)	100	100
Contamination	0.43	0.48

Completeness and contamination scores were calculated using CheckM (v. 1.1.2, default parameters) [51].

## Phylogenies based on multi-locus sequence and core-genome analyses

**Fig. 1.**
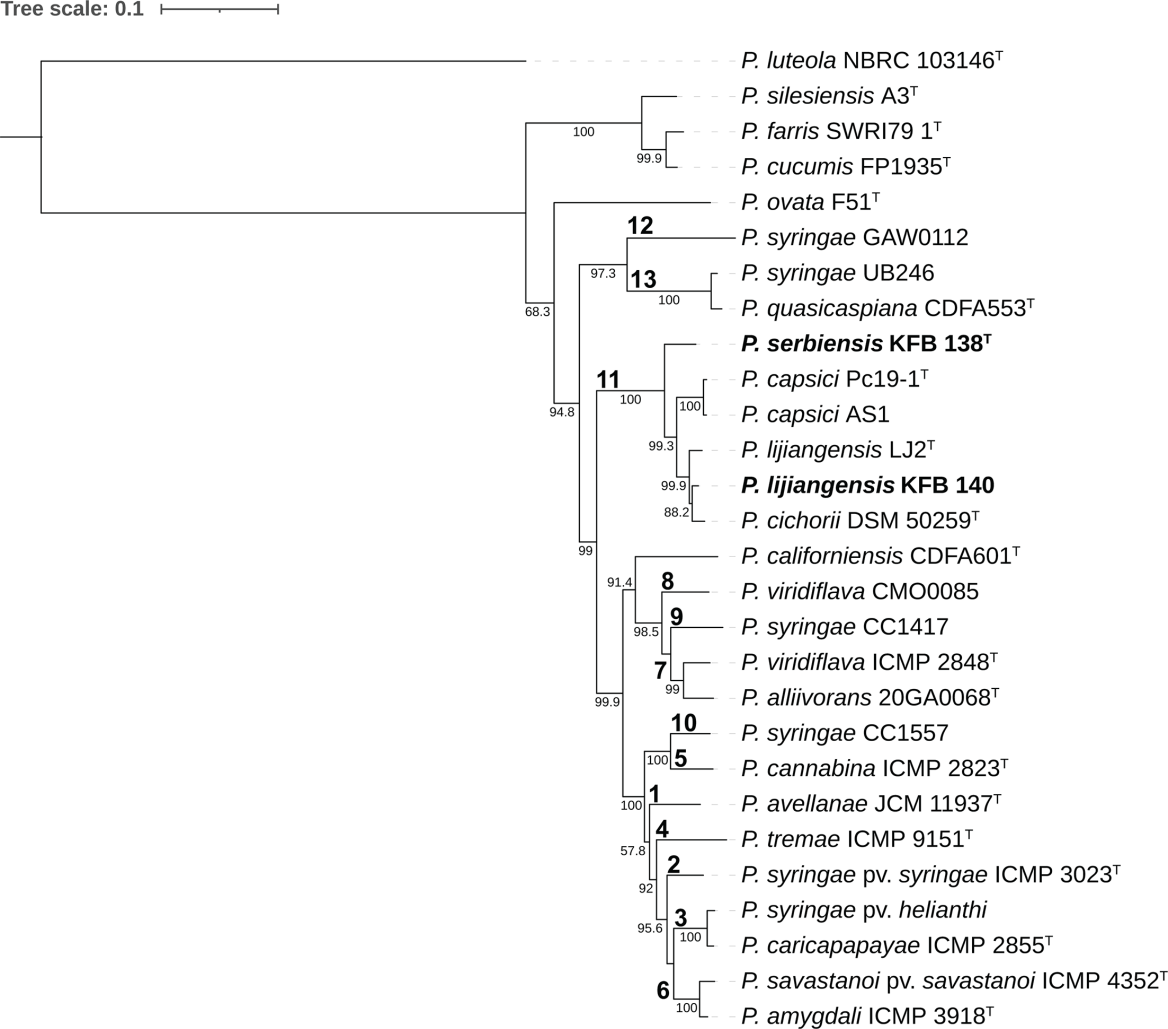
Maximum-likelihood phylogenetic tree based on concatenated alignments of housekeeping genes *gltA*, *rpoD*, *gapA* and *gyrB.* Bootstrap values based on 100 replicates are indicated at branching points. Bolded numbers designate Pssc phylogroups. The scale bar designates substitutions per site.

**Fig. 2.**
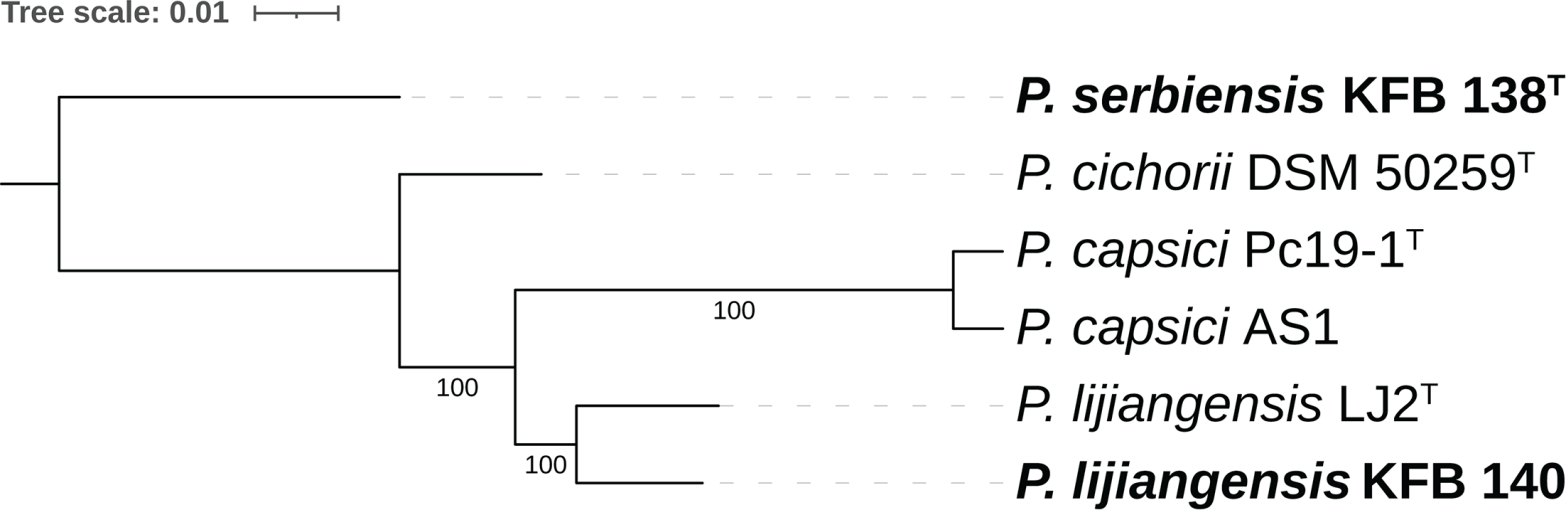
Maximum-likelihood phylogenetic tree based on the alignment of 2761 core genes of phylogroup 11 strains. Bootstrap values based on 100 replicates are indicated at branching points.

*Pseudomonas cichorii*, of which strains KFB 138 and KFB 140 had previously been described as members, is included within the *Pseudomonas syringae* species complex (Pssc), a large phylogenetic group that contains members of the species *P. syringae* as well as numerous other closely related *Pseudomonas* species, including many which have been documented to cause leaf spot symptoms on cucurbits [32,40, 41]. The species complex has been divided into 13 phylogroups based on MLSA of housekeeping genes *cts* (*gltA*), *rpoD*, *gapA* and *gyrB*, with *P. cichorii* being placed into phylogroup 11 [40]. To determine the phylogenetic placement of the Serbian strains, MLSA based on these four genes was conducted. Reference genomes from NCBI or gene sequences from the Plant Associated and Environmental Microbes Database (pamdb.org) were obtained for *Pseudomonas* strains representing the phylogroups of the Pssc, species outside of the complex, and those to which the isolates had the highest 16S rRNA sequence similarities. Analysis was performed using the program AutoMLSA2 (v. 0.8.1) [42] and a maximum-likelihood phylogenetic tree was generated using IQ-TREE (v. 2.1.3) [43] and visualized using ITOL (v. 6.9) [44] and Adobe Illustrator. Both KFB 138 and KFB 140 grouped most closely with Pssc phylogroup 11, alongside *P. cichorii* (strain DSM 50259^T^, accession number GCF_018343775.1), *P. capsici* (strain Pc19-1^T^, accession number GCF_017165765.1) and *P. lijiangensis* (strain LJ2^T^, accession number GCF_018968705.1) (Fig. 1). KFB 138 formed a lineage basal to all other phylogroup 11 members, while KFB 140 was placed into a clade along with *P. cichorii* DSM 50259^T^. This clade was sister to *P. lijiangensis* LJ2^T^. The nine *P. capsici* strains isolated from cucurbits in Alabama are represented in the phylogeny by strain AS1, which grouped closely with *P. capsici* type strain Pc19-1^T^. To further elucidate the phylogenetic relationship of phylogroup 11 members, their genomes were annotated using the programme Prokka (v. 1.14.6) [45] and then analysed with pan-genome pipeline tool Roary (v. 3.13.0) using default parameters, which identified a shared core genome of 2761 genes [46]. The resulting core-gene nucleotide alignment was then used to construct a maximum-likelihood phylogenetic tree as previously described (Fig. 2). In the core-genome phylogeny, * P. lijiangensis* LJ2^T^ and KFB 140 were placed into the same clade, distinct from *P. cichorii*. As seen in the MLSA phylogeny, KFB 138 formed a lineage basal to all other phylogroup 11 members.

## Whole-genome analyses

Whole-genome analyses, including ANIb (Table 3) and isDDH, were performed by comparing the genomes of the Serbian strains to those of reference strains to which they shared close phylogenetic relationships or high 16S rRNA sequence similarity. ANIb analysis was conducted using Python module pyani (v. 0.2.12, default parameters), while isDDH was calculated using the web tool genome-genome distance calculator (formula 2, v. 3.0, http://ggdc.dsmz.de/home.php) [47,49]. Strain KFB 138 was found to be most closely related to *P. cichorii* type strain DSM 50259^T^, to which it shared 91.20% ANIb and 44.6% isDDH. However, as both of these values are below the established thresholds for species identification (ANI: 95%; DDH: 70%) [50], KFB 138 was unable to be identified as a member of any existing *Pseudomonas* species to which it was compared. This is consistent with the phylogenetic placement of the strain into a clade distinct from all other *Pseudomonas* strains included in the analysis. Based on these results, we propose the creation of a new species within the genus *Pseudomonas*, designated *Pseudomonas serbiensis* (KFB 138^T^). ANIb analysis identified KFB 140 as a member of *P. lijiangensis* with 95.90% ANIb to type strain LJ2^T^ (Table 3). KFB 140 also had the highest isDDH similarity to *P. lijiangensis*, though with a value of 68.2%, which is below the accepted 70% threshold required for species determination [50]. The second highest isDDH value to KFB 140 was produced by *P. cichorii* DSM 50259^T^ and was significantly lower at 57.5%. While MLSA phylogeny placed strain KFB 140 in close proximity to *P. lijiangensis*, the strain had grouped most closely with *P. cichorii*. Though the two species are closely related, this result is not consistent with those produced by ANIb and isDDH, demonstrating the limitations of MLSA phylogenetic analysis for species identification. Core-genome analysis of phylogroup 11 members provided better resolution of the phylogenetic relationship of the strains and placed strains KFB 140 and *P. lijiangensis* LJ2^T^ within a single clade, separate from *P. cichorii*. Based on ANIb and core-genome phylogeny results, with supporting evidence from isDDH, KFB 140 was determined to belong to the species *P. lijiangensis*.

**Table 3.** Genomic relationships between strains included in this work (*P. serbiensis* sp. nov KFB 138^T^ and *P. lijiangensis* KFB 140) and type strains of closely related *Pseudomonas* species

*P. serbiensis* KFB 138**^T^**				
Species	NCBI accession	16S rRNA gene sequence similarity (%)	ANIb (%)	isDDH (%)
*P. cichorii* DSM 50259^T^	GCF_018343775.1	99.73	91.2	44.6
*P. lijiangensis* LJ2^T^	GCF_018968705.1	99.87	89.6	39.8
*P. lijiangensis* KFB 140	GCF_039503445.1	99.81	89.32	40.2
*P. capsici* Pc19-1^T^	GCF_017165765.1	99.07	89.08	38.2
*P. meliae* ICMP 6289^T^	GCF_001400515.1	98.34	80.62	24.9
*P. viridiflava* ICMP 2848^T^	GCF_001642795.1	97.39	80.57	24.9
*P. cerasi* 58^T^	GCF_900074915.1	97.56	80.49	24.9
*P. cannabina* ICMP 2823^T^	GCF_001400175.1	98.17	80.38	24.8
*P. alliivorans* 20GA0068^T^	GCF_017826695.1	98.1	80.31	24.6
*P. amygdali* ICMP 3918^T^	GCF_002699855.1	97.32	80.28	24.7
*P. savastanoi* ICMP 4352^T^	GCF_001401285.1	98.2	80.27	24.7
*P. congelans* DSM 14939^T^	GCF_900103225.1	98.37	80.05	24.4
*P. fragariae* 17^T^	GCF_026130225.1	98.24	80	24.6
*P. caricapapayae* ICMP 2855^T^	GCF_001400735.1	98.42	79.91	24.3
*P. syringae* ICMP 3023^T^	GCF_001401075.1	98.36	79.81	24.4
*P. californiensis* CDFA602^T^	GCF_021147775.1	98.13	79.72	24.2
*P. ovata* F51^T^	GCF_003131185.1	98.4	78.13	23.1
*P. quasicaspiana* CDFA550^T^	GCF_021147825.1	98.25	78.06	23.6
*P. lini* DSM 16768^T^	GCF_001042905.1	98.2	77.76	24.4
*P. farris* SWRI79^T^	GCF_019145235.1	98.25	76.96	23
*P. cucumis* FP1935^T^	GCF_030687935.1	98.18	76.95	22.4
*P. brassicae* MAFF 212427^T^	GCF_010671725.1	97.97	76.31	22.4
*P. laurentiana* JCM 32154^T^	GCF_014648275.1	96.89	75.88	21.8
***P. lijiangensis* KFB 140**				
**Species**	**NCBI accession**	**16S rRNA gene sequence similarity (%**)	**ANIb (%**)	**isDDH (%**)
*P. lijiangensis* LJ2^T^	GCF_018968705.1	99.87	95.9	68.2
*P. cichorii* DSM 50259^T^	GCF_018343775.1	99.73	94.0	57.5
*P. capsici* Pc19-1^T^	GCF_017165765.1	98.92	92.0	48.1
*P. viridiflava* ICMP 2848^T^	GCF_001642795.1	97.19	82.0	25.2
*P. asturiensis* LMG 26898^T^	GCF_900143095.1	96.94	81.9	25.1
*P. triticifolii* DOAB 1067^T^	GCF_014358015.1	96.5	81.9	25.2
*P. meliae* ICMP 6289^T^	GCF_001400515.1	98.27	81.8	24.9
*P. alliivorans* 20GA0068^T^	GCF_017826695.1	98.18	81.8	24.9
*P. amygdali* ICMP 3918^T^	GCF_002699855.1	97.12	81.6	24.8
*P. fragariae* 17^T^	GCF_026130225.1	98.04	81.5	24.9
*P. cannabina* ICMP 2823^T^	GCF_001400175.1	98.11	80.8	25.2
*P. savastanoi* ICMP 4352^T^	GCF_001401285.1	98.14	80.6	24.9
*P. congelans* DSM 14939^T^	GCF_900103225.1	98.17	80.5	24.9
*P. syringae* ICMP 3023^T^	GCF_001401075.1	98.15	80.2	24.8
*P. caricapapayae* ICMP 2855^T^	GCF_001400735.1	98.21	80.2	24.6
*P. lini* DSM 16768^T^	GCF_001042905.1	98.2	79.0	22.9
*P. brassicae* MAFF 212427^T^	GCF_010671725.1	98.11	78.6	22.4
*P. ovata* F51^T^	GCF_003131185.1	98.2	78.6	23.6
*P. quasicaspiana* CDFA550^T^	GCF_021147825.1	98.05	78.3	23.7
*P. laurentiana* JCM 32154^T^	GCF_014648275.1	96.82	78.0	22
*P. silesiensis* A3^T^	GCF_001661075.1	98.18	77.3	23.1
*P. cucumis* FP1935^T^	GCF_030687935.1	98.25	77.2	22.6
*P. farris* SWRI79^T^	GCF_019145235.1	98.31	77.2	22.7

## Comparison to TYGS

To further support the species designations of the two novel strains, their genomes were compared with the DSMZ’s Type Genome Server (TYGS), an online tool that compares user-uploaded genomes to a curated database of prokaryotic-type strain genomes using isDDH [48]. The TYGS was unable to identify either strain as a member of any prokaryotic species contained within its database; however, phylogenies produced by the server based on 16S rRNA gene sequences and whole-genome comparisons (Figs S1–S4, available in the online Supplementary Material) were consistent with those produced by previous MLSA and core-genome analyses, placing novel strains into a clade alongside type strains of *P. lijiangensis*, *P. cichorii* and *P. capsici*.

## Physiological and chemotaxonomic analyses

Further analyses were conducted to characterize strains KFB 138 and KFB 140 and support the creation of the novel species *P. serbiensis*. Cultures of strains were grown overnight in nutrient broth (Difco) before undergoing imaging at the University of Florida’s Interdisciplinary Center for Biotechnology Research with a Tecnai G2 Spirit TWIN 120 kV transmission electron microscope. From TEM images, cells of both strains were observed to be rod-shaped (Fig. S5).

Biochemical profiling of strains was conducted using the Biolog Gen III MicroPlate system (Table S1). Cultures were grown overnight on Biolog Universal Growth agar (Biolog Inc.), after which bacterial suspensions were created in Biolog inoculation fluid and adjusted to the recommended level of turbidity. One hundred microliters of suspension was added to each well of a MicroPlate; plates were then incubated at 28 °C for 24 h. Results were compared to the Biolog database (MicroLog™ M System, v. 5.1.1), which identified both strains as members of the genus *Pseudomonas*, though they could not be further identified as members of any existing species included in the database.

Bacterial cultures were sent to Charles River Laboratories (Newark, DE) for MALDI-TOF MS. Spectral results were compared to Bruker Biotyper (v. 11758) and Charles River spectral reference libraries (v. 23.01) for species identification. Charles River provides probable species identification for samples when a similarity score to a reference strain is produced which is greater than or equal to 1.75. Based on this, both *P. lijiangensis* KFB 140 and *P. serbiensis* sp. nov. KFB 138^T^ received probable identification as *P. cichorii*, with respective scores of 2.180 and 2.040, further illustrating the difficulty in differentiating the species through phenotypic means.

## Conclusions

In summary, multiphasic analyses, including 16S rRNA sequence analysis, phylogeny based on multi-locus sequence of four housekeeping genes, calculation of ANIb and *in silico* DDH values based on whole-genome sequences, biochemical profiling with the Biolog Gen III MicroPlate system and analysis with MALDI-TOF MS, were used for characterization and identification of bacterial isolates from Serbia, previously identified as *P. cichorii*, as members of *P. lijiangensis* as well as the proposed novel species *P. serbiensis*.

## Description of *Pseudomonas serbiensis* sp. nov

*Pseudomonas serbiensis* (ser.bi.en’sis. N.L. fem. adj. serbiensis, pertaining to Serbia).

Cells are Gram-negative rods (1.4–3.0 µm in length and 0.7–1.0 µm in width). Colonies are white to cream in coloration, smooth and circular, and 2.0–2.2 mm in diameter when grown on nutrient agar (Difco) and incubated for 48 h at 28 °C. Colonies produce a diffusible fluorescent pigment when grown on KMB. Type strain KFB 138^T^ is negative for levan production, pectolytic activity, and arginine dihydrolase activity and does not elicit an HR in tobacco. The strain is positive for oxidase activity and does elicit an HR in tomatoes. The strain was observed to grow when exposed to pH 5 and 6 as well as to salinity levels of 1 and 4% NaCl, though not at 8% NaCl. Inoculation of watermelon (*Citrullus lanatus*) and squash (*Cucurbita pepo* subsp. *pepo*) seedlings with KFB 138^T^ led to the development of severe disease symptoms. Growth of isolate KFB 138^T^ in tryptic soy broth (Sigma-Aldrich) was assessed at incubation temperatures of 0, 4, 15, 21, 28, 32, 37 and 41 °C. After 48 h of incubation, growth was observed only in cultures incubated at temperatures between 15 and 32 °C, with optimal growth occurring between 21 and 32 °C. Type strain KFB 138^T^ (NCPPB 4762^T^=LMG 33366^T^) was isolated from muskmelon (*Cucumis melo*) seedlings displaying stem rot and wilting in Srem, Serbia. KFB 138^T^ has a genome size of 6.22 Mb with a G+C content of 57.50%. The genome can be accessed in NCBI using GenBank accession number GCF_030580745.1; the 16S rRNA gene sequence accession number is OR725086.1.

## Supplementary material

10.1099/ijsem.0.006613 Uncited Supplementary Material 1.

## Abbreviations

ANI: average nucleotide identity
ANIb: average nucleotide identity based on blast
DDH: DNA–DNA hybridization
HR: hypersensitive response
isDDH: in silico DNA–DNA hybridization
KMB: King’s Medium B
MALDI-TOF MS: matrix-assisted laser desorption/ionization time of flight mass spectrometry
MLSA: multi-locus sequence analysis
NCBI: National Center for Biotechnology Information
Pssc: *Pseudomonas syringae* species complex
TEM: transmission electron microscopy
TYGS: Type Genome Server
