# Supplementary material for: Pseudomonas serbiensis sp. nov. isolated from watermelon and muskmelon in Serbia
Source: Int J Syst Evol Microbiol. 2024 Dec 18;74(12):006613. doi: 10.1099/ijsem.0.006613 (PMC12453560; doi:10.1099/ijsem.0.006613)
Supplement: Uncited Supplementary Material 1. [file ijsem-74-06613-s001.pdf]

Supplementary Figure 1. A 16S rRNA gene sequence phylogeny including *P. serbiensis* KFB-138^T and relevant *Pseudomonas* type strain genomes as produced by the online Type Genome Server (TYGS, https://tygs.dsmz.de/)

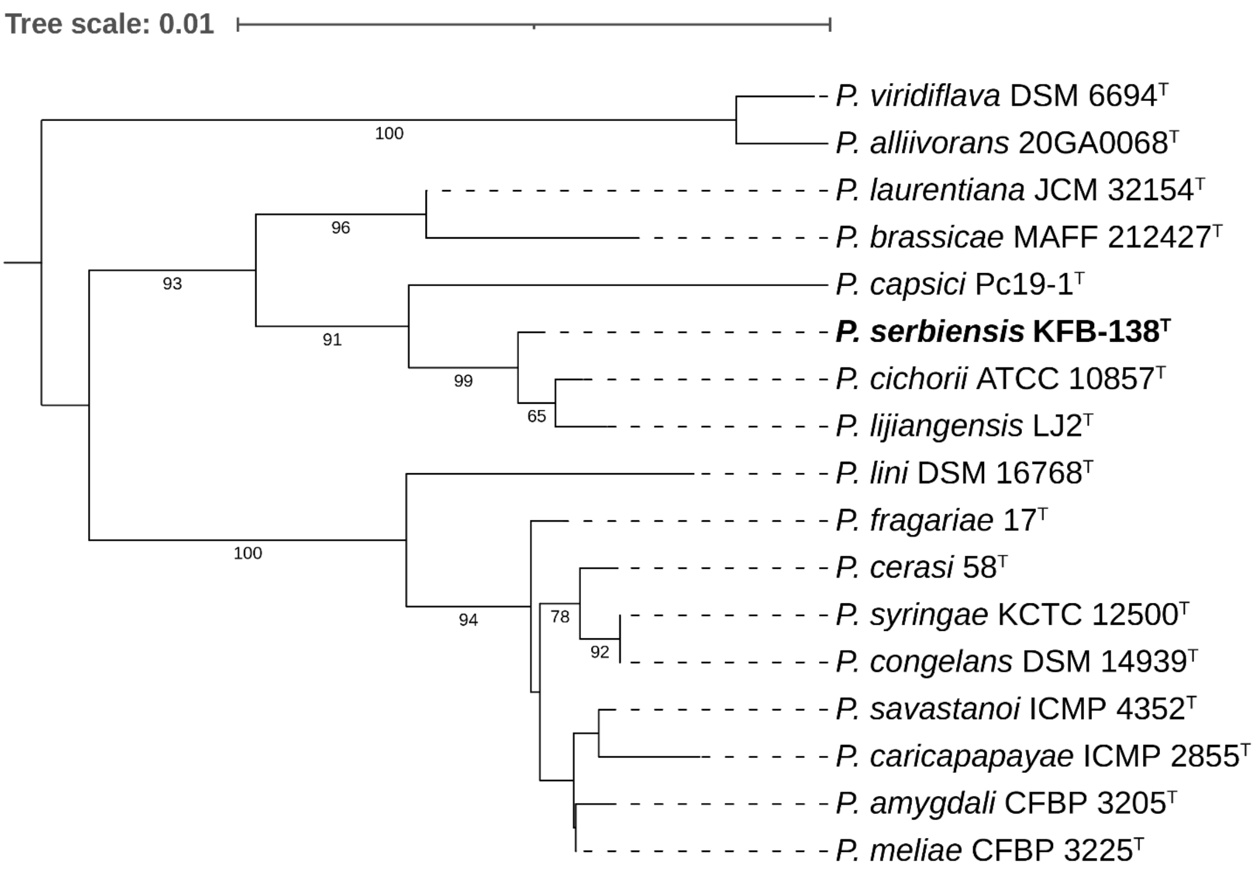

Phylogeny generated using distance formula 5 and distance algorithm CharacterCoverage.

Bootstrap values based on 100 replicates are indicated at branching points. The scale bar designates substitutions per site.

Supplementary Figure 2. A whole-genome phylogeny based on isDDH comparisons of *P. serbiensis* KFB-138^T and relevant *Pseudomonas* type strain genomes as produced by the online Type Genome Server (TYGS, https://tygs.dsmz.de/)

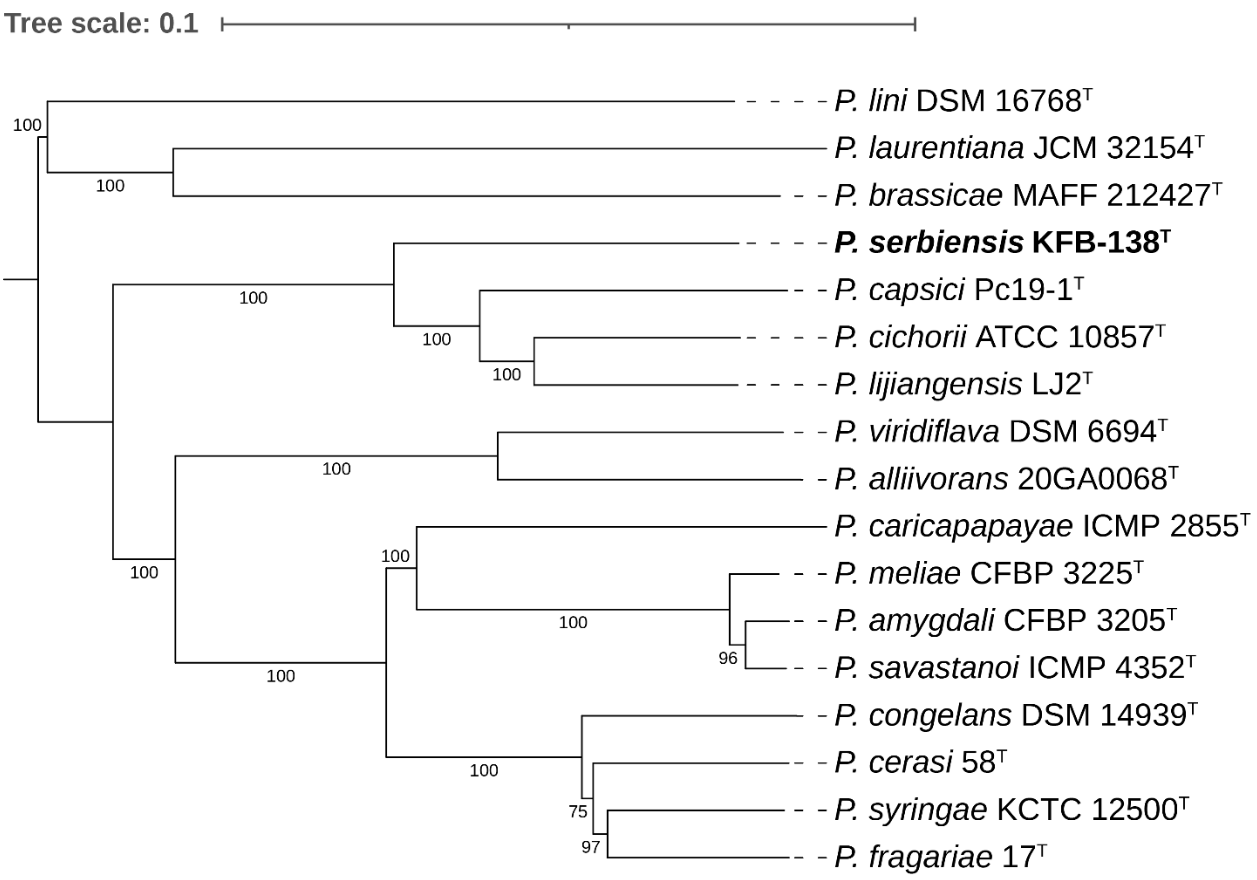

Phylogeny generated using distance formula 5 and distance algorithm GreedyWithTrimming.

Bootstrap values based on 100 replicates are indicated at branching points. The scale bar designates substitutions per site.

Supplementary Figure 3. A 16S rRNA gene sequence phylogeny including *P. lijiangensis* KFB-140 and relevant *Pseudomonas* type strain genomes as produced by the online Type Genome Server (TYGS, https://tygs.dsmz.de/)

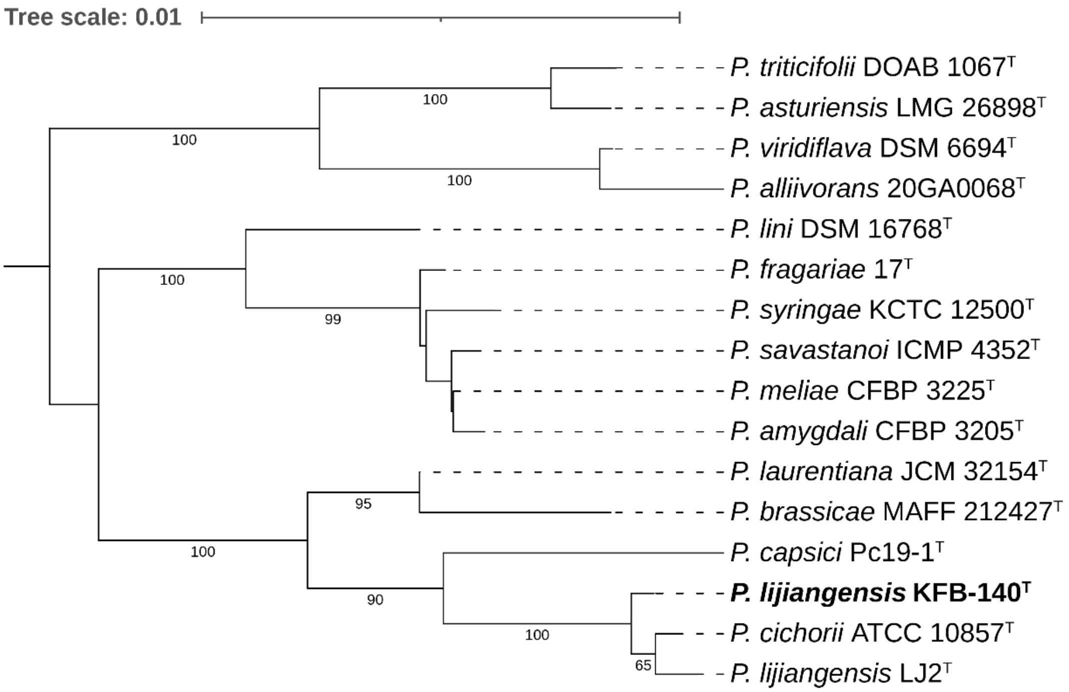

Phylogeny generated using distance formula 5 and distance algorithm CharacterCoverage.

Bootstrap values based on 100 replicates are indicated at branching points. The scale bar designates substitutions per site.

Supplementary Figure 4. A whole-genome phylogeny based on isDDH comparisons of *P. lijiangensis* KFB-140 and relevant *Pseudomonas* type strain genomes as produced by the online Type Genome Server (TYGS, https://tygs.dsmz.de/)

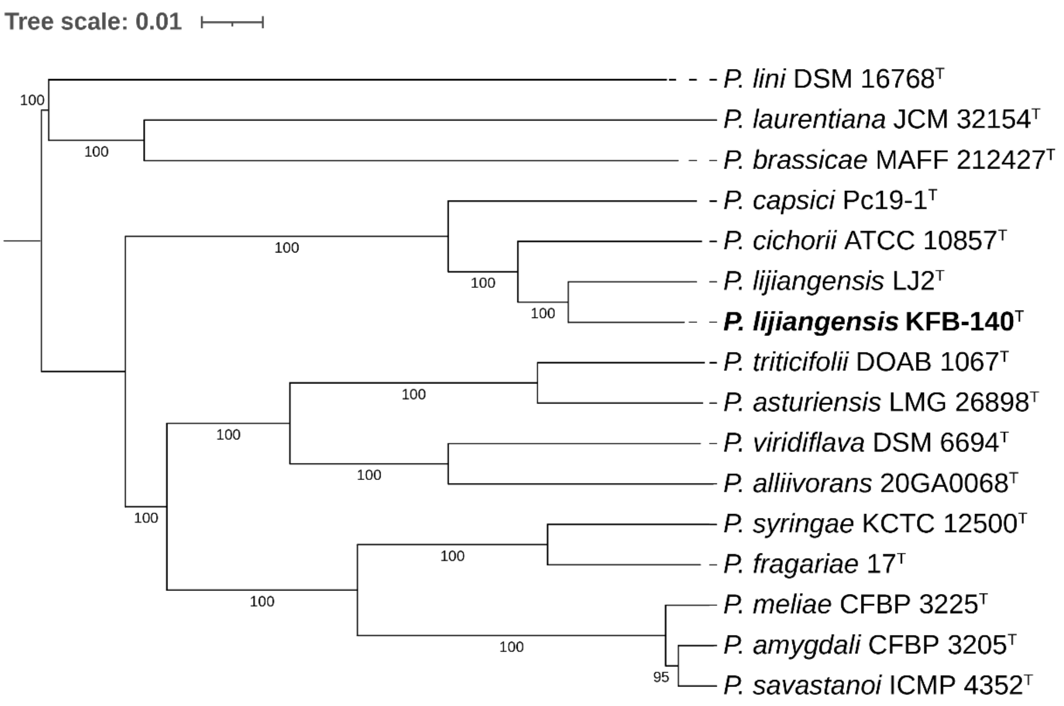

Phylogeny generated using distance formula 5 and distance algorithm GreedyWithTrimming.

Bootstrap values based on 100 replicates are indicated at branching points. The scale bar designates substitutions per site.

Supplementary Figure 5. Images of cells of (**A**) *Pseudomonas lijiangensis* KFB 140 and (**B**) *Pseudomonas serbiensis* sp. nov. KFB 138^{T} as captured by a Tecnai G2 Spirit TWIN 120 kV transmission electron microscope, University of Florida's Interdisciplinary Center for Biotechnology Research (ICBR)

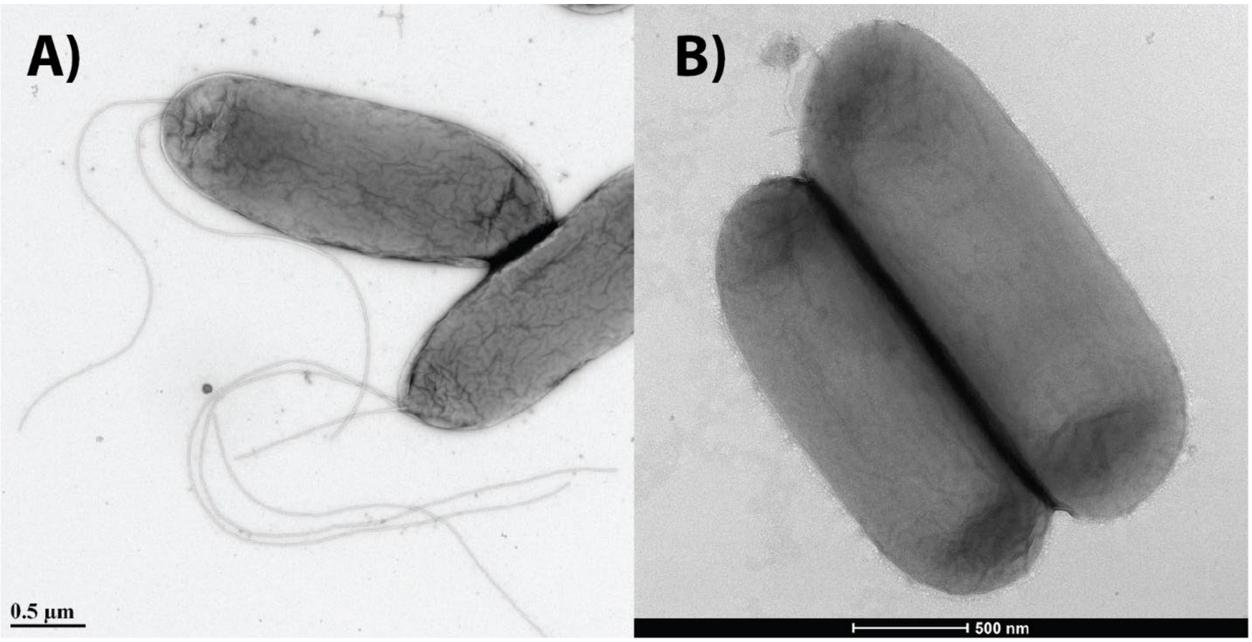

Supplementary Table 1. Biolog Gen III assay results for *P. serbiensis* sp. nov KFB 138^T, *P. lijiangensis* KFB 140, and relevant *Pseudomonas* type strains: **1**, *P. serbiens* sp. nov. KFB 138^T; **2**, *P. lijiangensis* KFB 140; **3**, *P. lijiangensis* LJ2^T; **4**, *P. cichorii* ATCC 10857^T; **5**, *P. capsici* Pc-19^T.

**Strain:**	**1**	**2**	**3**	**4**	**5**
**Source:**	This study	This study	Lu et al., 2022	Timilsina et al., 2018	Zhao et al., 2021
**Carbon Source Utilization:**					
Dextrin	-	-	NR	-	NR
D-Maltose	-	-	NR	-	NR
D-Trehalose	-	-	NR	-	NR
D-Cellobiose	-	-	NR	-	NR
Gentiobiose	-	-	-	-	NR
Sucrose	-	-	NR	-	-
D-Turanose	-	-	NR	-	NR
Stachyose	-	+	NR	-	NR
D-Raffinose	-	-	NR	-	NR
α-D-Lactose	-	-	NR	-	NR
D-Melibiose	-	-	NR	-	NR
β-Methyl-D-Glucoside	-	-	NR	-	NR
D-Salicin	-	-	NR	-	NR
N-Acetyl-D-Glucosamine	-	-	NR	-	NR
N-Acetyl-β-D-Mannosamine	-	-	NR	-	NR
N-Acetyl-D-Galactosamine	-	-	NR	-	NR
N-Acetyl Neuraminic Acid	-	+	NR	-	NR
α-D-Glucose	+	+	+	+	NR
D-Mannose	+	+	+	+/-	NR
D-Fructose	+	+	NR	+	NR
D-Galactose	+	+	NR	+	NR
3-Methyl Glucose	-	-	NR	-	NR
D-Fucose	+	+	NR	-	+
L-Fucose	-	+/-	NR	-	NR
L-Rhamnose	-	-	NR	-	NR
Inosine	+	+	NR	+	NR
D-Sorbitol	+/-	-	NR	-	-
D-Mannitol	-	-	NR	+	NR
D-Arabitol	-	-	NR	+	NR
myo-Inositol	+/-	-	NR	+	NR
Glycerol	+/-	-	NR	+	NR
D-Glucose-6-PO_4	-	-	NR	-	NR

Supplementary Table 1 Continued

Strain:	1	2	3	4	5
Source:	This study	This study	Lu et al., 2022	Timilsina et al., 2018	Zhao et al., 2021
**Carbon Source Utilization:**					
D-Fructose-6-PO_4	+	+	+	-	NR
D-Aspartic Acid	+	+	NR	+	NR
D-Serine	+	+	+/-	-	NR
Gelatin	-	-	NR	-	NR
Glycyl-L-Proline	-	-	NR	+/-	NR
L-Alanine	+	+	NR	+/-	NR
L-Arginine	-	-	NR	+	NR
L-Aspartic Acid	+	+	NR	+	NR
L-Glutamic Acid	+	+	NR	+	NR
L-Histidine	-	-	NR	+	NR
L-Pyroglutamic Acid	+	-	NR	+	NR
L-Serine	+	+	NR	+/-	NR
Pectin	-	-	NR	-	NR
D-Galacturonic Acid	-	+/-	-	-	NR
L-Galacturonic Acid	+/-	-	NR	-	NR
D-Gluconic Acid	-	-	+	+	NR
D-Glucuronic Acid	+	+/-	NR	-	NR
Glucuronamide	+	+	+	-	NR
Mucic Acid	+	+	NR	+/-	NR
Quinic Acid	+	+	NR	+/-	NR
D-Saccharic Acid	+	+	NR	+/-	NR
p-Hydroxy-Phenylacetic Acid	-	-	NR	-	NR
Methyl Pyruvate	-	-	NR	-	NR
D-Lactic Acid Methyl Ester	-	-	NR	-	NR
L-Lactic Acid	+	+	+	+	NR
Citric Acid	+	+	+	+/-	NR
α-Keto-Glutaric Acid	+	+	+	+	NR
D-Malic Acid	+	+	+	+/-	NR
L-Malic Acid	+	+	+	+/-	NR
Bromo-Succinic Acid	-	+	NR	-	NR
Tween 40	+/-	-	-	-	NR
γ-Amino-Butyric Acid	+	+/-	NR	+	NR
α-Hydroxy-Butyric Acid	-	-	NR	-	NR
β-Hydroxy-D,L-Butyric Acid	-	-	NR	+	NR
α-Keto-Butyric Acid	-	-	NR	-	NR
Acetoacetic Acid	-	-	NR	-	NR
Propionic Acid	-	-	NR	+	NR
Acetic Acid	+	+	+	+/-	NR
Formic Acid	-	+/-	NR	-	NR

Supplementary Table 1 Continued

Strain:	1	2	3	4	5
Source:	This study	This study	Lu et al., 2022	Timilsina et al., 2018	Zhao et al., 2021
**Chemical Sensitivity Assays:**					
pH 6	+	+	+	+	NR
pH 5	+	+	NR	+	NR
1% NaCl	+	+	+	+	NR
4% NaCl	+	+	+	+	NR
8% NaCl	+	-	NR	-	NR
1% Sodium Lactate	+	+	+	+	NR
Fusidic Acid	+	+	NR	+	NR
D-Serine	+	+	+	-	NR
Troleandomycin	+	+	+	-	+
Rifamycin SV	+	+	+	+	NR
Minocycline	-	-	NR	-	NR
Lincomycin	+	+	+	+	NR
Guanadine HCl	+	+	+	+/-	NR
Niaproof 4	+	+	+	+	NR
Vancomycin	+	+	+	+	NR
Tetrazolium Violet	+	+	+	+	NR
Tetrazolium Blue	+	+	NR	+	NR
Nalidixic Acid	+	+	NR	+	NR
Lithium Chloride	+/-	+	-	-	NR
Potassium Tellurite	+	+	NR	+	NR
Aztreonam	+	+	+	+/-	NR
Sodium Butyrate	-	-	NR	+/-	NR
Sodium Bromate	-	-	NR	+	NR

Positive reactions are designated (+); negative reactions are designated (-); indeterminate reactions are designated (+/-)
